# Delirium diagnosis without a gold standard: Evaluating diagnostic accuracy of combined delirium assessment tools

**DOI:** 10.1371/journal.pone.0267110

**Published:** 2022-04-18

**Authors:** Stephana J. Moss, Chel Hee Lee, Christopher J. Doig, Liam Whalen-Browne, Henry T. Stelfox, Kirsten M. Fiest

**Affiliations:** 1 Department of Community Health Sciences, Cumming School of Medicine, University of Calgary, Calgary, Alberta, Canada; 2 Department of Critical Care Medicine, University of Calgary and Alberta Health Services, Calgary, Alberta, Canada; 3 O’Brien Institute for Public Health, University of Calgary, Calgary, Alberta, Canada; 4 Hotchkiss Brain Institute, University of Calgary, Calgary, Alberta, Canada; 5 Alberta Health Services, Calgary, Alberta, Canada; 6 Department of Psychiatry, Cumming School of Medicine, University of Calgary, Calgary, Alberta, Canada; University of Florence, ITALY

## Abstract

**Background:**

Fluctuating course of delirium and complexities of ICU care mean delirium symptoms are hard to identify or commonly confused with other disorders. Delirium is difficult to diagnose, and clinicians and researchers may combine assessments from multiple tools. We evaluated diagnostic accuracy of different combinations of delirium assessments performed in each enrolled patient.

**Methods:**

Data were obtained from a previously conducted cross-sectional study. Eligible adult patients who remained admitted to ICU for >24 hours with at least one family member present were consecutively enrolled as patient-family dyads. Clinical delirium assessments (Intensive Care Delirium Screening Checklist [ICSDC] and Confusion Assessment Method-ICU [CAM-ICU]) were completed twice daily by bedside nurse or trained research assistant, respectively. Family delirium assessments (Family Confusion Assessment Method and Sour Seven) were completed once daily by family members. We pooled all delirium assessment tools in a single two-class latent model and pairwise (i.e., combined, clinical or family assessments) Bayesian analyses.

**Results:**

Seventy-three patient-family dyads were included. Among clinical delirium assessments, the ICDSC had lower sensitivity (0.72; 95% Bayesian Credible [BC] interval 0.54–0.92) and higher specificity (0.90; 95%BC, 0.82–0.97) using Bayesian analyses compared to pooled latent class analysis and CAM-ICU had higher sensitivity (0.90; 95%BC, 0.70–1.00) and higher specificity (0.94; 95%BC, 0.80–1.00). Among family delirium assessments, the Family Confusion Assessment Method had higher sensitivity (0.83; 95%BC, 0.71–0.92) and higher specificity (0.93; 95%BC, 0.84–0.98) using Bayesian analyses compared to pooled latent class analysis and the Sour Seven had higher specificity (0.85; 95%BC, 0.67–0.99) but lower sensitivity (0.64; 95%BC 0.47–0.82).

**Conclusions:**

Results from delirium assessment tools are often combined owing to imperfect reference standards for delirium measurement. Pairwise Bayesian analyses that explicitly accounted for each tool’s (performed within same patient) prior sensitivity and specificity indicate that two combined clinical or two combined family delirium assessment tools have fair diagnostic accuracy.

## Background

One third of patients admitted to the intensive care unit (ICU) will develop delirium [1, 2] that is characterized by acute onset and a fluctuating course of symptoms that are hard to identify or commonly confused with other disorders [3]. Delirium is often underdiagnosed and unmanaged [4] despite several delirium assessment tools available for use by both the patient care team and visiting family members [5]. Family members know a patient best and engaging them in delirium detection may help to make earlier note of unrecognized patient changes [6].

A systematic review and psychometric analysis on five clinical delirium assessment tools (i.e., administered by a trained clinical/research observer) from 36 patient cohorts reported the Intensive Care Delirium Screening Checklist (ICDSC) and Confusion Assessment Method-ICU (CAM-ICU) had the highest validity and reliability among critically ill adults [7]. Family delirium assessments (i.e., administered by a family member or friend) have fair, but lower, diagnostic accuracy in critically ill adults in comparison, and in addition to, clinical delirium assessments [8].

The diagnostic accuracy of a delirium assessment tool is determined by comparing the results from a delirium assessment to a gold standard test [with 100% accuracy] [9]. There is no gold standard delirium diagnostic tool and available reference standards are imperfect owing to the fluctuating course of delirium and complexities associated with ICU care [10]. This often leads researchers and patient care teams to combine results from several delirium assessments to increase accuracy of diagnosis [11]. Most literature on this topic reports sensitivity and specificity estimates from combinations of delirium assessments performed in different ICU patients [7].

Latent class models are employed widely to estimate diagnostic accuracy without a gold standard [12] as this approach does not require a reference standard be selected arbitrarily as a criterion standard, which is considered more realistic and reflective of practice [13]. Latent class analyses rely on the central assumption of conditional independence [14]. Another approach is pairwise Bayesian analyses that estimates the probability of an event based on prior knowledge of conditions that are related to the event, common in psychology to improve diagnostic accuracy. This approach can explicitly account for prior uncertainty in the sensitivity and specificity for each individual diagnostic tool [15]. The objective of this study was to use latent class models and pairwise Bayesian analyses to evaluate diagnostic accuracy of two combined clinical or two combined family delirium assessment tools used within the same critically ill adult patient.

## Methods

### Study design and setting

This diagnostic evaluation study used data from a previously published cross-sectional study [8] and is reported according to Strengthening the Reporting of Observational Studies in Epidemiology (STROBE) guidelines for cross-sectional studies (S1 Table).

### Participants

Recruitment of participants was performed at a large, tertiary care academic hospital (Foothills Medical Centre [FMC], Calgary, Canada) within a single-payer health system. A multidisciplinary care team staffs the 28 closed beds of the FMC medical-surgical ICU. Eligibility criteria for study participation are listed in S2 Table. Eligibility for participation was assessed daily by a trained research assistant following approval to approach a family granted by the bedside nurse. Patients who met eligibility criteria with at least one present family member (e.g., spouse, child, friend) who provided informed consent were consecutively enrolled as a patient-family dyad.

### Procedure

The study collected data in ICU up to a maximum of five days. Patient and family demographics (e.g., age, sex) were collected at first delirium assessment. Patient clinical characteristics (e.g., admitting diagnosis, Acute Physiology and Chronic Health Evaluation-II [APACHE-II]) were obtained from eCritical, a beside clinical information system validated for research purposes [16].

### Measures

#### Clinical delirium assessments

Two clinical delirium assessments were completed by assessors (i.e., nurse or research assistant) blind to the results of the family administered delirium assessments. The ICDSC was conducted twice daily (once per 12-hour nursing shift; standard of care) and is valid and reliable to screen for delirium on eight independent domains (i.e., hallucinations/delusions/psychosis, level of consciousness, inattention, disorientation, psychomotor agitation, inappropriate speech or mood, sleep wake/cycle disturbance, and symptom fluctuations) [17]. ICDSC delirium assessments yield an ordinal score ranging from 0 to 8 that can be dichotomized to classify patients consistent with delirium (score of ≥4) or not having delirium (score of 0 to 3) [18]. 64% sensitivity and 90% specificity are reported for ICDSC assessments for this sample of patient-family dyads [8]. The second clinical assessment was the four-item dichotomous CAM-ICU (i.e., scored “delirium present” or “delirium absent”), bid (9:00AM-11:00AM; 2:00PM-4:00PM) on all eligible patients by a trained research assistant. The CAM-ICU has 76% sensitivity and 83% specificity in this sample [8].

#### Family administered delirium assessments

Two family administered delirium assessments were performed once daily by family members of critically ill patients blind to results from clinical assessments. Family members assessed patient delirium using the Family Confusion Assessment Method (FAM-CAM) and the Sour Seven. Using the FAM-CAM, family members answered 11-items regarding sudden changes to patient attention, orientation, perception or concentration [19]. The FAM-CAM is a dichotomous (i.e., scored “delirium present” or “delirium absent”) family-administered delirium assessment tool with 54% sensitivity and 77% specificity in this sample [8]. The Sour Seven was also used to assess patient delirium symptoms related to altered awareness, disordered thinking, and reduced attention [20]. The Sour Seven is scored out of 18 with a cutpoint ≥4 (i.e., indicating probable delirium); a cutpoint score ≥4 in this sample has 73% sensitivity and 69% specificity [8].

### Data analysis

Data are presented as numbers/percentage, mean or median. Measures of diagnostic accuracy (e.g., sensitivity, specificity) are reported with accompanying 95% Confidence Intervals (CIs) or 95% Bayesian Credible (BC) intervals, as appropriate. From the primary study sample (of 147 dyads) we included 73 patient-family dyads that contributed one set of data for *both* pairwise assessments within 6-hour time windows, chosen to account for the fluctuation in delirium presentation [3]. To be included in analyses, each patient-family dyad needed to have a CAM-ICU, FAM-CAM *and* Sour Seven that were all recorded within 6-hours of when the ICDSC (i.e., standard of care) was performed (i.e., pairwise clinical assessments [ICDSC + CAM-ICU] and pairwise family assessments [FAM-CAM + Sour Seven], with each delirium assessment performed no longer than 6 hours after the ICDSC) [21]. Each dyad contributed data for one pair of clinical delirium assessments and one pair of family delirium assessment; when multiple pairs of data for a single dyad were reported, the pair that reported the most severe scores was used [22]. We used complete case analysis and no imputation techniques for missing data were employed. Statistical analyses were conducted in SAS 9.4 (SAS Institute Inc.), STATA ICV.16 (StataCorp LLC), and R (R Core Team, 2020). The poLCA (Polytomous Variable Latent Class Analysis) package was used to conduct latent class analysis [23] and the WinBUGS statistical software was used for Bayesian analysis [24–26].

We employed two statistical techniques to evaluate diagnostic accuracy of combined delirium assessments, accounting for each tool’s prior sensitivity and specificity, and with the novelty of being used within the same critically ill patient [26–29]. For the first technique, we used latent class analysis [27, 28] that pooled all four delirium assessments (i.e., ICDSC + CAM-ICU + FAM-CAM + Sour Seven) to simultaneously estimate the sensitivity and specificity for each individual assessment tool. To satisfy the central assumption for the present analyses to report estimates of diagnostic accuracy, all four (clinical and family) delirium assessments were pooled. A two-class model was selected based on the lowest Bayesian Information Criterion (S3 Table). Results from latent class analyses should be interpreted as the diagnostic criteria for an individual delirium assessment tool if that tool were to be used alone, without considering each tool’s prior estimated diagnostic criteria (i.e., sensitivity and specificity) [30].

We used pairwise Bayesian analyses for combined delirium assessments (i.e., pairwise clinical assessments, ICDSC + CAM-ICU; or pairwise family assessments, FAM-CAM + Sour Seven) [26, 29]. We chose these combinations *a priori*, considering our potential conclusions as combination of multiple clinical *or* multiple family assessment tools is more practical, and feasible. Using the BUGS software [24], random samples were drawn using Monte Carlo Markov Chain considering posterior distributions of parameters. We report summary statistics based on these random samples for which convergence was ensured. We ran 15,000 iterations of the Gibbs sampler; three different chains were generated, and we followed the Gelman-Rubin’s diagnostic to approximate convergence [31]. The first 5,000 iterations were discarded to report posterior summaries. Results from Bayesian analyses should be interpreted as diagnostic accuracy of combined delirium assessment tools all used within the same critically ill adult patient, with consideration of each tool’s prior estimated diagnostic criteria.

### Ethical approval

The study was approved by the Conjoint Health Research Ethics Board at the University of Calgary (REB 16–2060).

## Results

### Population characteristics

**Fig 1:**
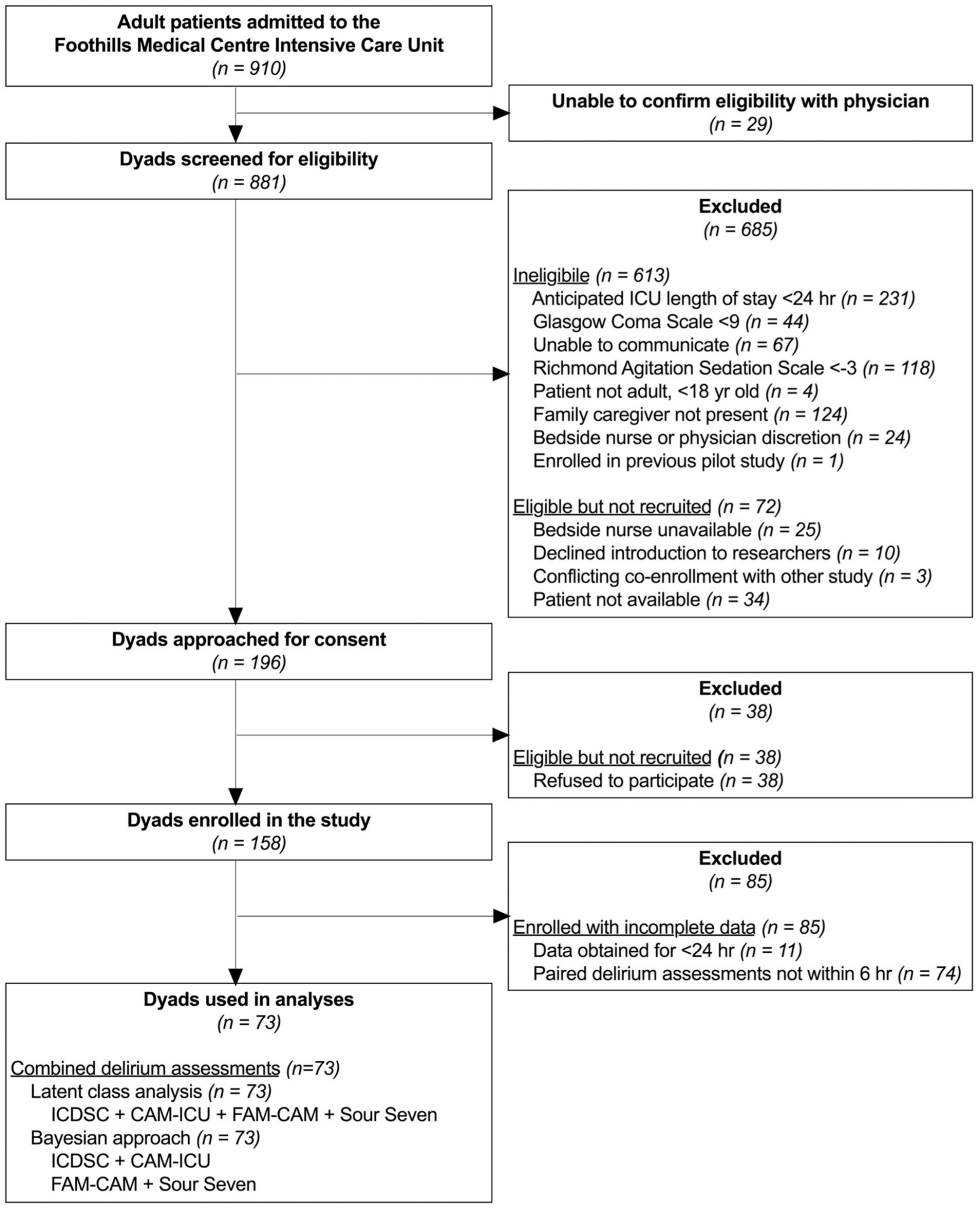
Participant flow diagram.

A total of 73 patient-family member dyads from the full study sample [8] were included in these analyses (Fig 1). We excluded 85/147 dyads that did not provide data suitable to conduct pairwise Bayesian estimates (i.e., dyads that did not have the CAM-ICU, FAM-CAM *and* Sour Seven all recorded within 6-hours of the ICDSC). Demographic and clinical characteristics of the 73 included patient-family member dyads are shown in Table 1. Critically ill patients were primarily admitted with a medical diagnosis (n = 35, 48.0%) and were on average 57.8 years (SD, 15.8 yr); 67% (n = 49) of patients were male. Family members were on average 53.5 years (SD 14.7 yr) and mostly (n = 61, 83.6%) female. Majority (n = 45, 61.6%) of family members reported having completed some university/college education, or greater. The median Richmond Agitation Sedation-Scale Score at the time of ICDSC assessment that was included in analysis was 0 (interquartile range, -1 to 0) (i.e., “alert and calm”).

**Table 1:** Demographic and clinical characteristics of 73 included patients and family members.

Characteristic	Dyads		
	Adult Patient (*n* = 73)	Family Member (*n* = 73)
Age, yr, mean (SD)	57.78 (15.76)	53.45 (14.71)
Sex, female, *n* (%)	24 (30.38)	61 (83.56)
Education, *n* (%)		
High school or less	33 (45.21)	28 (38.36)
Some university/college or greater	40 (54.79)	45 (61.64)
Patient admitting diagnosis category, *n* (%)		
Medical	35 (47.95)	--
Neurologic	14 (19.18)	--
Trauma	13 (17.81)	--
Surgical	11 (15.07)	--
Acute Physiology and Chronic Health Evaluation II score, median (IQR)	21 (15–26)	--
Richmond Agitation-Sedation Scale, median (IQR)^a^	0 (-1-0)	--

IQR = interquartile range, SD = standard deviation

^a^At the timepoint of ICDSC delirium assessment included in analysis

Dashes indicate no data to report for that group

### Results of delirium assessments

Results of patient delirium assessments are presented in Table 2. On average, clinical delirium assessment tools were performed 1.83 hours (SD 1.54) apart. Majority (n = 40, 54.8%) of patients were classified “without delirium” by both clinical assessments for delirium (i.e., ICDSC and CAM-ICU). Many patients (n = 30, 41.1%) had conflicting assessment results between the CAM-ICU and ICDSC. Only 4.1% (n = 3) of patients were classified “with delirium” by both clinical assessments; common in our cohort of patients when less than clinical threshold symptoms of delirium are presented (not identified by the CAM-ICU, but ICDSC) [32]. Family delirium assessments using the FAM-CAM and Sour Seven which were done by the same family member at the same time, demonstrated more consistent results than clinical assessments. Twenty-eight patients (38.4%) were classified “without delirium” and 23 patients (31.5%) were classified “with delirium” by both the FAM-CAM and Sour Seven conducted within a 6-hour window.

**Table 2:** Patient delirium assessment results, paired.

**Clinical Assessments**			
**CAM-ICU** ^b^		**ICDSC** ^a^	
		**With Delirium (*n* = 12)**	**Without Delirium (*n* = 61)**
		***n* (%)**	***n* (%)**
**With Delirium**	**(*n* = 24)**	3 (4.11)	21 (28.77)
**Without Delirium**	**(*n* = 49)**	9 (12.33)	40 (54.79)
**Family Assessments**			
**Sour Seven** ^d^		**FAM-CAM** ^c^	
		**With Delirium (*n* = 37)**	**Without Delirium (*n* = 36)**
		***n* (%)**	***n* (%)**
**With Delirium**	**(*n* = 31)**	23 (31.51)	8 (10.96)
**Without Delirium**	**(*n* = 42)**	14 (19.18)	28 (38.36)

CAM-ICU = Confusion Assessment Method for ICU, FAM-CAM = Family Confusion Assessment Method, ICDSC = Intensive Care Delirium Screening Checklist

^a^ICDSC is scored out of 8; cutpoint of 4

^b^CAM-ICU is scored out of 7; cutpoint of 3

^c^FAM-CAM is scored as present/absent

^d^Sour Seven is scored out of 18; cutpoint of 4

### Diagnostic accuracy from latent class analysis

Estimates of diagnostic accuracy from a two-class latent model are shown in Table 3. Among clinical assessments the ICDSC had lower sensitivity (ICDSC, 0.79 [95% CI 0.58–0.93]; CAM-ICU, 0.88 [95% CI 0.68–0.97]) and negative predictive value (ICDSC, 0.91 [95% CI 0.80–0.97]; CAM-ICU, 0.93 [95% CI 0.81–0.99]) than the CAM-ICU. In contrast, the ICDSC had higher specificity (ICDSC, 1.00 [95% CI 0.93–1.00]; CAM-ICU, 0.82 [95% CI 0.68–0.91]) and positive predictive value (ICDSC, 1.00 [95% CI 0.83–1.00]; CAM-ICU, 0.70 [95% CI 0.51–0.85]) than the CAM-ICU.

**Table 3:** Diagnostic criteria for clinical and family delirium assessments from latent class analysis for pooled, whole sample (*n* = 73).

Assessments	Sensitivity		Specificity		PPV		NPV	
	Mean	95% CI	Mean	95% CI	Mean	95% CI	Mean	95% CI
*Clinical*								
ICDSC^a^	0.79	0.58–0.93	1.00	0.93–1.00	1.00	0.83–1.00	0.91	0.80–0.97
CAM-ICU^b^	0.88	0.68–0.97	0.82	0.68–0.91	0.70	0.51–0.85	0.93	0.81–0.99
*Family*								
FAM-CAM^c^	0.79	0.58–0.93	0.63	0.48–0.77	0.51	0.34–0.68	0.86	0.71–0.96
Sour Seven^d^	0.67	0.45–0.84	0.69	0.55–0.82	0.53	0.33–0.70	0.81	0.66–0.91

CAM-ICU = Confusion Assessment Method for ICU, FAM-CAM = Family Confusion Assessment Method, ICDSC = Intensive Care Delirium Screening Checklist, NPV = Negative Predictive Value, PPV = Positive Predictive Value

^a^ICDSC is scored out of 8; cutpoint of 4

^b^CAM-ICU is scored out of 7; cutpoint of 3

^c^FAM-CAM is scored as present/absent

^d^Sour Seven is scored out of 18; cutpoint of 4

Latent class analysis evaluates each delirium assessment tool after pooling together all four tools without considering each tool’s sensitivity and specificity.

Diagnostic accuracy estimated from latent class analysis for family delirium assessments were lower than clinical delirium assessments. Sensitivity of the FAM-CAM and Sour Seven were 0.79 (95% CI 0.58–0.93) and 0.67 (95% CI 0.45–0.84), respectively. Specificity estimates were 0.63 (95% CI 0.48–0.77) and 0.69 (95% CI 0.55–0.83) for the FAM-CAM and Sour Seven, respectively. Positive predictive values (FAM-CAM, 0.51 [95% CI 0.34–0.68]; Sour Seven, 0.53 [95% CI 0.33–0.70]) were lower than negative predictive values (FAM-CAM, 0.86 [95% CI 0.71–0.96]; Sour Seven, 0.81 [95% CI 0.66–0.91]).

### Diagnostic accuracy from Bayesian analyses

Estimates for sensitivity and specificity from pairwise Bayesian analyses are shown in Table 4. In combining results from clinical delirium assessments, the ICDSC had lower sensitivity (0.72 [95% BC 0.54–0.92]) and specificity (0.90 [95% BC 0.82–0.97]) than the CAM-ICU (sensitivity, 0.92 [95% BC 0.70–1.00]; specificity, 0.94 [95% BC 0.80–1.00]). In combining results from family delirium assessments, the FAM-CAM had higher sensitivity and specificity compared to the Sour Seven. Sensitivity estimates were 0.83 (95% BC 0.73–0.92) and 0.64 (95% BC 0.47–0.82) and specificity estimates were 0.93 (95% BC 0.84–0.98) and 0.85 (95% BC 0.67–0.99) for the FAM-CAM and Sour Seven, respectively.

**Table 4:** Diagnostic criteria for clinical and family delirium assessments from pairwise Bayesian analyses for whole sample (*n* = 73).

	**Pairwise Clinical Assessments**							
	**ICDSC** ^a^					**CAM-ICU** ^b^		
	**Sensitivity**		**Specificity**			**Sensitivity**		**Specificity**
	**Mean**	**95% BC**	**Mean**	**95% BC**	**Mean**	**95% BC**	**Mean**	**95% BC**
*DIC = 18*.*80*	0.72	0.54–0.92	0.90	0.82–0.97	0.92	0.70–1.00	0.94	0.80–1.00
	**Pairwise Family Assessments**							
	**FAM-CAM** ^c^					**Sour Seven** ^d^		
	**Sensitivity**		**Specificity**			**Sensitivity**		**Specificity**
	**Mean**	**95% BC**	**Mean**	**95% BC**	**Mean**	**95% BC**	**Mean**	**95% BC**
*DIC = 17*.*95*	0.83	0.73–0.92	0.93	0.84–0.98	0.64	0.47–0.82	0.85	0.67–0.99

BC = Bayesian Credible, CAM-ICU = Confusion Assessment Method for ICU, DIC = Deviance Information Criterion, FAM-CAM = Family Confusion Assessment Method, ICDSC = Intensive Care Delirium Screening Checklist, NPV = Negative Predictive Value, PPV = Positive Predictive Value

^a^ICDSC is scored out of 8; cutpoint of 4

^b^CAM-ICU is scored out of 7; cutpoint of 3

^c^FAM-CAM is scored as present/absent

^d^Sour Seven is scored out of 18; cutpoint of 4

Bayesian analysis evaluates each delirium assessment tool in a pairwise manner and considers each tool’s sensitivity and specificity.

## Discussion

The motivation for this study was to employ two statistical techniques to evaluate diagnostic accuracy of combined clinical or family delirium assessment tools all used within the same critically ill adult patient. The first technique, latent class analysis, assessed performance characteristics (i.e., sensitivity, specificity) without being informed by each tool’s prior estimated results. In contrast, Bayesian analysis specifically accounted for each tool’s prior performance characteristics in estimating post-test probabilities or performance. Both analyses performed comparably and suggest that two combined clinical or two combined family delirium assessment tools have fair diagnostic accuracy.

A gold standard tool [test with 100% diagnostic accuracy] is not available for delirium detection and the development of such a test is likely not feasible or practical [26, 33]. In the delirium literature it is recommend using either the 10th revision of the International Statistical Classification of Diseases and Related Health Problems (ICD-10) or the Diagnostic and Statistical Manual of Mental Disorders, 5th Edition (DSM-5) as the reference standard, as applied by trained clinicians [34]. It is common to see reference to earlier DSM versions (e.g., 4th Edition, DSM-4) as they are considered easier to operationalize than the DSM-5 [35]; the Confusion Assessment Method (CAM) was developed based on the DSM-3-R as a user-friendly diagnostic tool to screen for delirium in most clinical populations [36]. Lack of gold standard and plethora of available (but imperfect) reference standards [5] means combinations of results from many delirium assessment tools are used to determine if delirium is present.

Thomas et al. [37] referred to the DSM-5 to estimate diagnostic accuracy compared to a combination of the ICD-10 and the CAM in a sample of hospitalized elderly patients, and reported higher sensitivity and specificity for the CAM when used in combination with the ICD-10. Shenkin et al. [11] assessed diagnostic accuracy of the 4 A’s Test (4AT)—a short delirium assessment tool—and the CAM against the DSM-4 in a sample of older acute medical inpatients. The 4AT had 76% sensitivity and 94% specificity while the CAM had 40% sensitivity and 100% specificity. Since no delirium assessment tool is perfect [10], by arbitrarily selecting an imperfect delirium reference it is impossible to account for the true uncertainty in estimated diagnostic criteria for delirium assessments.

Latent class analysis is frequently used to estimate diagnostic accuracy from imperfect diagnostic tests [27, 28]. This statistical technique does not require arbitrary selection of a reference standard and is thought to be more realistic and reflective of practice. One limitation of this approach is that latent class models assume conditional independence of test results within each patient given the latent true delirium status—referred to as the central assumption of conditional independence (i.e., test results are independent given latent classes). Latent class analysis also relies on the central assumption of conditional independence [14]. To satisfy this assumption, we pooled all delirium assessment tools in a single two-class latent model, which prevented considering each tool’s prior estimated diagnostic criteria.

Our results are in line with our earlier report [8] such that for delirium diagnosis in ICU, a balance of sensitivity and specificity in diagnostic accuracy should be considered given that delirium diagnosis in critically ill patients is often subjective and challenging [4]. For example, in clinical practice administration of a pharmacological treatment requires high specificity (few false positive delirium cases) whereas for screening purposes high sensitivity (few false negative delirium cases) is desired.

We have previously shown feasibility and acceptability of employing family administered delirium assessment tools in adult ICU [38], and reported that diagnostic accuracy from family delirium assessments are still fair, but lower, in comparison and in addition to clinical delirium assessments [8]. In the present study we found that combining results from family assessments had higher sensitivity and specificity compared to either the FAM-CAM or Sour Seven used alone, which were also higher compared to estimates reported from our earlier diagnostic accuracy study in this sample [8]. While we do not propose that family members can accurately “diagnose” delirium it is possible that engaging family members in delirium detection might be helpful to identify earlier symptoms. Future research should map items across the FAM-CAM and Sour Seven to create a single, family tool with maximized sensitivity and specificity and validate this tool in a large sample of patients.

The results of our study must be interpreted cautiously and there are several limitations worth noting. First, this study was conducted as a secondary analysis of data collected in an earlier observational study. Second, our data was collected from patients admitted to a single medical center. Although this center serves a large catchment area of approximately 1.8 million people and should be reasonably representative of multisystem ICU patients in academic hospitals, these finding may not be generalizable to all ICU patients. Third, nearly 20% of our sample were patients with a neurological admission status in whom delirium diagnosis is especially challenging as neurologic patients fall more under the term acute encephalopathy rather than delirium [39]. Our ability to disentangle what is acute encephalopathy from delirium in the acutely neurologically injured patient using the GCS was limited; increased false positives in our sample would have resulted in reduced diagnostic accuracy. Fourth, achieving repeated assessments over five days was challenging as family members were variably present at the bedside; this limited our sample size given that we included only dyads with results from delirium assessments recorded no longer than 6 hours apart. Fifth, we compared different diagnostic accuracy estimates from diverse statistical techniques that variably considered underlying uncertainly in the accuracy of different delirium assessment tools. Our results indicated that combined delirium assessments tools are more accurate; overestimation is possible considering our small sample size. New, combined versions of clinical and family delirium assessment tools require development, testing, and validation to provide additional evidence to substantiate our hypothesis.

## Conclusions

Caring for critically ill patients includes multiple tools to identify ICU delirium. Results from delirium assessment tools employed at different times during patient care are often combined owing to no gold standard and imperfect reference standards for delirium but using several tools at a single time is impractical and infeasible. Using pairwise Bayesian analyses to explicitly account for each tool’s prior sensitivity and specificity all in the same patient within 6-hours, we report that two combined clinical or two combined family delirium assessment tools have fair diagnostic accuracy.

## Supporting information

S1 Table STROBE statement; checklist of items that should be included in reports of *cross-sectional studies*. (DOCX)

S2 Table Study eligibility criteria. (DOCX)

S3 Table Summary of information criteria and estimated and observed probabilities of patient delirium using latent class analysis with a two-class model. (DOCX)
