# Supplementary material for: Delirium diagnosis without a gold standard: Evaluating diagnostic accuracy of combined delirium assessment tools
Source: PLoS One. 2022 Apr 18;17(4):e0267110. doi: 10.1371/journal.pone.0267110 (PMC9015135; doi:10.1371/journal.pone.0267110)
Supplement: S2 Table — (DOCX) [file pone.0267110.s002.docx]

**Supplemental Table 2.** Study eligibility criteria

| **Inclusion criteria** |
| --- |
| Age 18 years or older  Family member present  Richmond Agitation-Sedation Scale score <-3 (eligible for delirium detection) |
| **Exclusion criteria** |
| Glasgow Coma Scale score <9  Anticipated to have an ICU length of stay <24 hr  Patient or family did not provide informed consent  New primary neurologic injury (e.g., severe traumatic brain injury)  Unable to communicate with research staff (e.g., hearing impairment, not fluent in English) |
