# Supplementary material for: Delirium diagnosis without a gold standard: Evaluating diagnostic accuracy of combined delirium assessment tools
Source: PLoS One. 2022 Apr 18;17(4):e0267110. doi: 10.1371/journal.pone.0267110 (PMC9015135; doi:10.1371/journal.pone.0267110)
Supplement: S3 Table — (DOCX) [file pone.0267110.s003.docx]

**Supplement Table 3.** Summary of information criteria and estimated and observed probabilities of patient delirium using latent class analysis with a two-class model

|  |  | **Unobserved States to Select Number of Classes in Prediction Model** | | | | | | | | | |
| --- | --- | --- | --- | --- | --- | --- | --- | --- | --- | --- | --- |
| **Fitting Criteria** |  | **1** |  | **2^*^** | |  | | **3** |  | **4** |  |
| Bayesian Information Criteria |  | 400.46 |  | 392.86 | |  | | 397.97 |  | 414.02 |  |
| Akaike Information Criteria |  | 391.30 |  | 371.25 | |  | | 365.90 |  | 370.50 |  |
| **Delirium Assessment Results** |  | **Observed Patients with Delirium** | | |  | | **Expected Patients with Delirium^*^** | | | | |
| **(ICDSC^a^, CAM-ICU^b^, Sour Seven^c^, FAM-CAM^d^)** |  | **%** | | |  | | **%** | | | | |
| (0, 0, 0, 0) |  | 18.00 | | |  | | 16.42 | | | | |
| (0, 0, 0, 1) |  | 9.00 | | |  | | 10.55 | | | | |
| (0, 0, 1, 0) |  | 4.00 | | |  | | 7.60 | | | | |
| (0, 0, 1, 1) |  | 9.00 | | |  | | 5.17 | | | | |
| (0, 1, 0, 0) |  | 7.00 | | |  | | 4.33 | | | | |
| (0, 1, 1, 0) |  | 2.00 | | |  | | 2.61 | | | | |
| (0, 1, 1, 1) |  | 5.00 | | |  | | 3.51 | | | | |
| (1, 0, 1, 1) |  | 3.00 | | |  | | 1.52 | | | | |
| (1, 1, 0, 0) |  | 3.00 | | |  | | 1.51 | | | | |
| (1, 1, 0, 1) |  | 5.00 | | |  | | 4.20 | | | | |
| (1, 1, 1, 0) |  | 2.00 | | |  | | 2.65 | | | | |
| (1, 1, 1, 1) |  | 6.00 | | |  | | 7.38 | | | | |

CAM-ICU = Confusion Assessment Method for ICU, FAM-CAM = Family Confusion Assessment Method, ICDSC = Intensive Care Delirium Screening Checklist

^*^Two-class model selected to compute expected patients with delirium

^a^ICDSC is scored out of 8; cutpoint of 4

^b^CAM-ICU is scored out of 7; cutpoint of 3

^c^Sour Seven is scored out of 18; cutpoint of 9

^d^FAM-CAM is scored as present/absent
